# Carbon Reservoirs in Temperate South American Nothofagus Forests

**DOI:** 10.1100/tsw.2002.75

**Published:** 2002-01-08

**Authors:** Klaus Baswald, Jose D. Lencinas, Gabriel Loguercio

**Affiliations:** Factor Consulting and Management AG, Binzstrasse 18, 8045 Zürich, Switzerland; Centro Investigacion y Extension Forestal Andino Patagonico (CIEFAP), Ruta 259, km4, CC 14, 9200 Esquel, Chubut, Argentina

**Keywords:** C-reservoirs, forest ecosystems, South American forest ecosystems, Lenga

## Abstract

Humans are influencing the global carbon (C) cycle due to the combustion of fossil fuels and due to changes in land use management. These activities are fostering the manmade greenhouse effect and thus global climate change. Negative effects for life on earth are accounted for.

Among others the international climate debate focused attention on forests and forestry, knowing about their considerable influence on global climate change. Whilst the global C budget is described fairly well, there is a lack of regional data describing the C reservoirs and flows in detail. This has to be constituted especially for forests in developing countries.

This paper presents an investigation at regional scale of the C reservoirs in a South American forest ecosystem. The investigation puts emphasis on the area and stand volume estimation and the development of expansion and reduction factors. Vegetation types are classified and stratified, determining the corresponding areas and estimating the stand volume. Converting factors are developed to calculate C in branches and roots as a percentage of standing wood measured by inventories.

## INTRODUCTION

The global carbon (C) budget includes the atmosphere, the terrestrial biosphere (including soils), and the oceans. The largest C amounts of the terrestrial biosphere are stored in forests. About 80% of the C exchange between the atmosphere and the terrestrial biosphere are due to the buildup and decay of biomass in forests. Forests are therefore the main component in the earths' natural C cycle.

Mankind is influencing the global C cycle due to the combustion of fossil fuels and due to changes in land use management[1]. These activities are fostering the manmade greenhouse effect forcing global climate change. Negative effects for life on earth are accounted for.

Knowing about their considerable influence on global climate change, the international climate debate focused attention on forests and forestry, among other things. Although the global C budget is described fairly well, there is a lack of regional data describing the C reservoirs and flows in detail[2,3,4]. Most of the large-scale estimations are based on a restricted number of ecosystems and ecosystem type data[5,6,7,8]. This has to be constituted especially for forests in developing countries[9]. To close this data gap, there is a need to gain as many quantitative estimates of the biophysical and biochemical properties of forests as possible[10].

This paper presents an investigation at regional scale of the C reservoirs in a South American forest ecosystem on which there is little information available. The investigation puts emphasis on the area and stand volume estimation, and the development of expansion and reduction factors. Therefore, vegetation types are classified and stratified, determining the corresponding areas and estimating the stand volume. Converting factors are developed to calculate C in branches and roots as a percentage of standing wood measured by inventories.

## THE PROJECT AREA

The investigated area corresponds largely to the water catchment area of lakes La Plata and Fontana situated in the southern part of Argentina about 2000 km southwest of Buenos Aires in the Department of Rio Senguer, Province of Chubut, Patagonia. The area lies between 44°44’ and 45°03’ south, and 71°20’ and 72°04’ west on the eastern side of the Andes. It stretches 57 km from east to west and 20 km on average from north to south (Fig. 1).

The lakes La Plata and Fontana drain into the River Senguer, which rises here. The western, northern, and southern borders of the investigated area are defined by the watershed, at the same time the political border with Chile. The eastern border is formed by a fictitious line running north-south, level with the point at which Lake Fontana drains into the River Senguer. The investigated area thus covers a total area of 125,590 ha (1256 km$^2$), about 50,000 ha of which are forest. The surface of the two most important lakes covers 15,540 ha[11].

The water catchment area of the lakes comprises a unified socio-economic and ecologic region within the Department Alto Rio Senguer. The municipality of Rio Senguer, which is situated 70 km east of lake Fontana, has about 2000 inhabitants. At the height of the season there are about 100 to 200 permanent residents in the catchment area of the lakes. They earn their income in two small sawmills, on three Estanzias, in tourism, in a stone factory, and as frontier guards and foresters. A further 200 to 300 people are living on five large and ten small Estanzias with seasonal variations. Besides, some hundred tourists fill the area during summer. The main reasons for the touristic activities are the beauty of the landscape, and fishing and hunting activities to an increasing degree. All the indications are that the touristic activities will expand in the future. The western part of the project area belongs to the Municipality of Alto Rio Senguer, whereas the usufructs in this part belong to the Province of Chubut. The land in the eastern part of the project area belongs to the property of five large Estanzias[12].

Many inhabitants are seasonal workers for sheep shearing, with no employment in the rest of the year. Currently, there are about ten workers per Estanzia with seasonal fluctuations. That is half the number of employees the Estanzias had 20 years ago. The mean income of people in the project area is difficult to determine. A manager of an Estanzia might earn approximately $1000US per month, a worker may have $500US per month to his disposal. The returns of the Estanzias have been reduced in the last decades due to falling market prices for wool and an overgrazing of the pastures. Currently, to cover the living costs of a family of four, an Estanzia of about 20,000 ha is necessary.

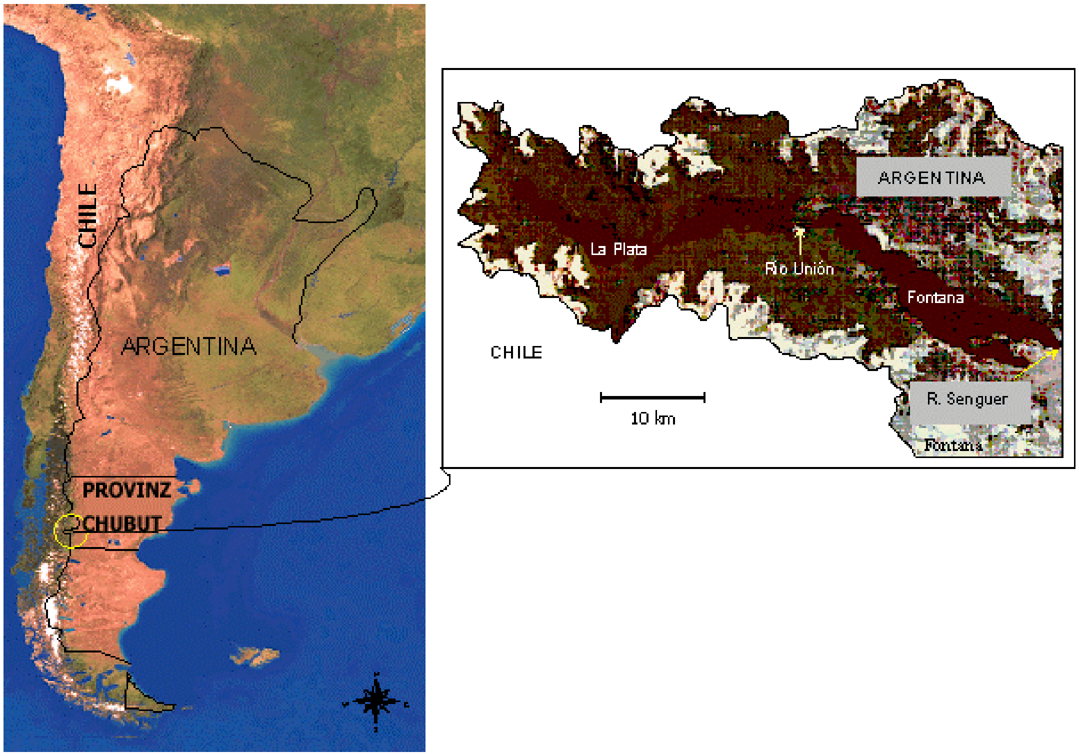

**FIGURE 1**. Position of the investigated area in Argentinian Patagonia. Left: Satellite picture, Planetary Visions 2000 (political borders are represented roughly); right: Landsat 5 TM (3,2,1) of 07.03.1999.

The main tree species in the investigated area is Lenga (*Nothofagus pumilio*), which appears in pure stands from the lakeshore up to the tree line. In areas sheltered from the wind, the Lenga reaches upper heights of 24 to 26 m, and in snowy areas with very strong wind at the tree line it forms scrub forests. Lenga produces a beautiful reddish-grained wood that is comparable to that of cherry (*Prunus avium*). It is well suited for furniture construction. Ñire stands (*N. antarctica*) are mainly situated on soil with a poor nutrient supply or shallow soil in lower areas, or Ñire appears in succession on formerly burnt areas[13].

The relief of the investigated area is mountainous and rugged. The main ridge of the Andes with the highest summits runs along the west of the project area, over the mountains Cerro Dedo (2020 m), north of Lake La Plata, and Cerro Catedral (2062 m) in the south.

In the project area the precipitation falls mainly as snow, and varies significantly from about 2500 mm/year in the western part of the project area to 500 mm/year in the eastern part[14].

The area is mainly inaccessible. There are two main roads along the north and south banks of the lakes as far as the River Unión, which are only passable with a cross-country vehicle up to the middle section of Lake La Plata. In addition there are some forest roads in poor condition at the lakes. They were built provisionally just for felling and skidding work and were not maintained when this was finished.

## METHODOLOGY

### Data Description

The SPOT HRVIR XI (multispectral mode) and P (monochromatic mode) were recorded by Kiruna on 1$^{st}$ January 1999. A summer picture was requested to optimize the spectral contrast between vegetation and other surfaces such as bare soils and water, and to minimize the topographic influence on the illumination. In order to estimate the area of vegetation burnt in January/February 1999, a Landsat 5 TM by CONAE (Argentina) was acquired. For georeferencing the satellite data, control points were located using GPS in differential mode. A digital elevation model was generated from topographical maps at a scale of 1:100 000[11].

Several software-based image analysis systems and program packages were used in the course of this study such as ERDAS Imagine 8.3, ArcView GIS 3.1, ARC/INFO 7.2, Pathfinder of Geoexplorer II. The statistical analysis was performed using SAS (Statistical Analysis System). For data assessment and processing in the estimation of the biomass in standing wood and main branches larger than 10 cm in diameter, Corel OCR-Trace 8 and ArcView GIS 3.0a software were used.

## Classification of the Vegetation

A combination of maximum likelihood[15] and binary hierarchical classification was used in this study. The vegetation-covering layer resulted from multiplying the SPOT-XI data by the water and vegetation masks (TVI index). The vegetation covering was then segmented into forest (Lenga and Ñire stands) and grassy areas with the maximum likelihood classifier (Fig. 2).

After the boundary of the forest area had been established, the altitude was included as additional information, derived from the digital elevation model, in order to avoid confusing Ñire and Lenga scrub (Lenga in higher areas) when considering only the spectral profile. The subsequent classification into Lenga and Ñire forest was performed using a synthetic band difference. In essence, two classes of Lenga stands were formed: potential production forests (altitude below 1150 m a.s.l.) and protection forests (altitude over 1150 m a.s.l. or growing in areas with >60% slope).

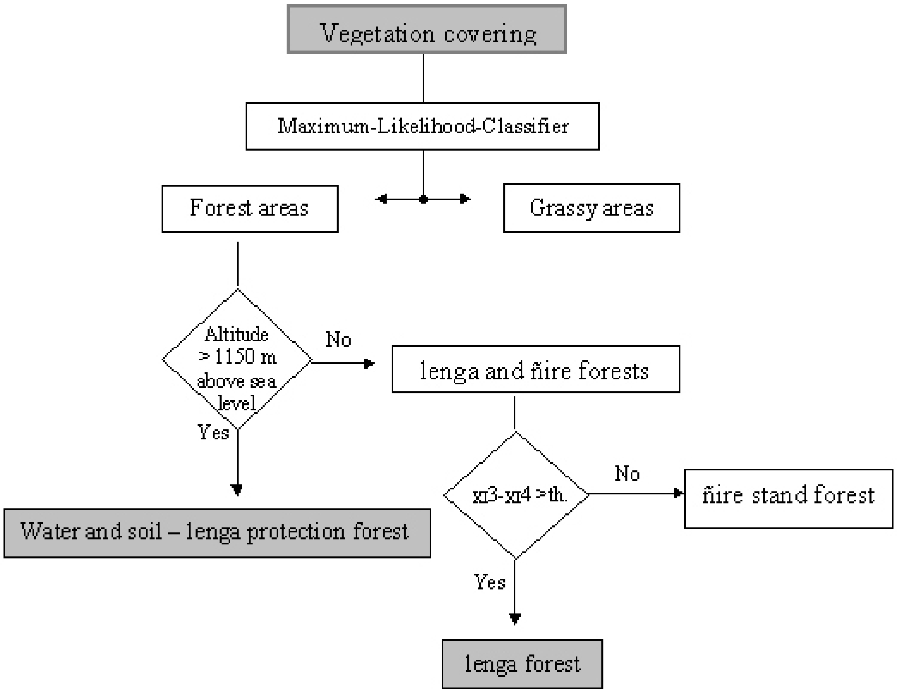

**FIGURE 2**. Overview of the classification process. th.: threshold.

## Estimation of Lenga Stand Volume

Remote sensing with satellite data has been used to record large forested areas and additional information for forest inventories since 1972. The rapid development of modern recording systems allowed for the effective and cost-efficient use of satellite data to classify forest types (among others: [16,17]) and for recording the state of the forest, as well as for investigating the parameters of forest stands[18,19,20,21,22].

For reasons of efficiency, remote sensing data have been used in combination with terrestrial samples for forest inventories in multiphase sampling methods since the beginning of the 1980s for large areas and rough terrain[23]. The two-phase sampling method is, for example, frequently applied in conjunction with aerial photography[24,25,26,27] and in recent years with digital satellite data[28,29]. In the first phase, the auxiliary variables are determined on the aerial or satellite sampling plots. In the second phase, a terrestrial subsample is taken and the variables of interest are measured terrestrially as accurately as possible. In the two-phase sampling with regression, the corresponding data from the first and second phase are used for the regression model developments, which are then used in the estimation of stand variables[30].

The use of two-phase sampling is justified if the costs per unit in the first sample are lower than those in the second phase, and if the precision required can be attained when the total sample size of both the first (calculated in units of second sample) and second sample is smaller than the sample size needed for the same precision when employing ground measurements only[31]. The critical value of the cost ratio is calculated as follows:

$$K = \frac{c_2}{c_1} > \frac{r^2}{\left(1 - \sqrt{1 - r^2}\right)^2}$$

where $c_1$ = cost per sample unit in phase 1, $c_2$ = cost per sample unit in phase 2, and r = correlation coefficient.

For simple regression, an approximation of the variance of the estimated mean of the variable of interest ($S_{\bar{y}}$) in the two-phase sampling is the following[32]:

$$S_{\bar{y}}^2 = \frac{S_y^2\left(1 - r^2\right)}{n_t} + \frac{r^2 s_y^2}{n_s}$$

where $S_y^2$ = variance of dependent variable in 2nd phase, $n_t$ = size of sample in 2nd phase, $n_S$ = size of sample in 1st phase, r = correlation coefficient.

This formula is valid for small values of $1/n_S$, which is true for large area forest inventories[31]. For multiple regression models the variance estimation method of Khan and Tripathi[33] is suitable.

## Definition of the Area to be Inventoried and Two-Phase Sampling Design

The class of potential Lenga production forest formed in the course of satellite data classification grows at approximately 920 to 1150 m a.s.l. To increase the accuracy of the stand volume estimate, and therefore the estimate of the C reservoirs of living trees, this class was subdivided further into two large strata for the forest inventory, taking information on land use, from a land register, and forest fires into account. The two Lenga forest types fulfill the following criteria (Table 1).

**TABLE 1**
**Criteria for Distinguishing Lenga Forest Types**

| | |
|---|---|
| Lenga forest **(stratum I)** | Lenga forest without restricting factors for the establishment of natural regeneration |
| Degraded Lenga forest **(stratum II)** | Lenga forest with hindrances to the establishment of natural regeneration (forest pasture and grazing damage, forest fires and low amount of precipitation) |
| Mean height of stand | >14 m |
| Slope | <60% |
| Altitude | <1150 m |

In this study, the following steps of the two-phase sampling method were used with the combination of satellite data and terrestrial measurements:

- Phase 1: a sample was set up systematically using a grid with a mesh size of 500 m on the satellite picture. From satellite data the reflection values from a 5 × 5 pixel window of the SPOT picture were extracted here as an independent variable. In this the quotients IR II [(XI3-XI4)/(XI3+XI4)] and IR [(XI3/XI2)] were calculated. These have already proven worthwhile in these questions.
- Phase 2: in a supporting sample taken terrestrially, those forest parameters (DBH, height) were recorded which, according to well-known dendrometric procedures, allow a precise estimate of the target variable (stand volume per hectare). The sample units are **permanent cluster samples** from five circular sample plots, each of 500 m² (Fig. 3).

This cluster sample was designed, bearing the following aspects in mind: time for the establishment and recording of a lump sample, and the analysis of the intracluster correlation coefficient[11].

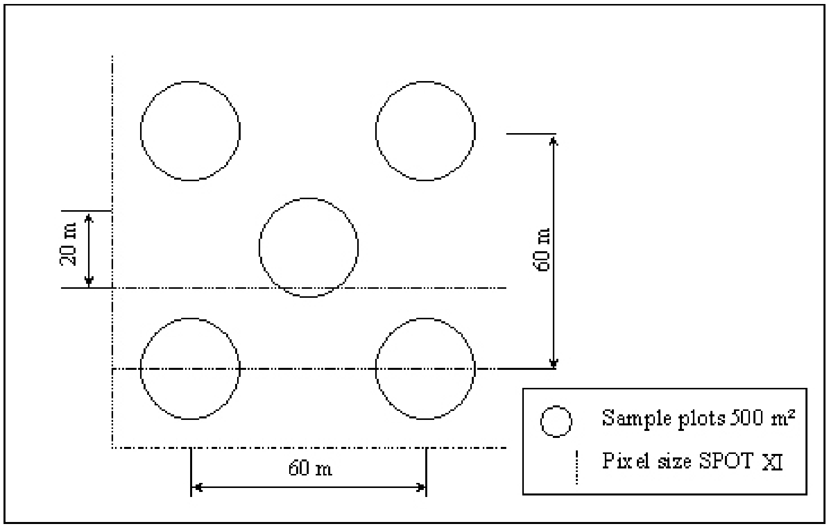

**FIGURE 3**. Cluster samples consisting of five circular plots of 500 m² each and their relation to the spatial resolution of the SPOT data.

The estimated values of the populations of both strata may be derived from the following equations[34]:

Mean stand volume per hectare in cluster I

$$\bar{y}_i = \frac{\sum_{j=1}^{M_i} y_{i,j}}{M_i}$$

Mean stand volume per hectare over all clusters

$$\bar{y} = \frac{\sum_{i=1}^{n} \bar{y}_{i,j}}{n}$$

and if $M_i$ varies among clusters using the ratio estimate

$$\bar{y} = \frac{\sum_{i=1}^{n} M_i \cdot \bar{y}_i}{\sum_{I=1}^{n} M_i} = \frac{\sum_{i=1}^{n} \sum_{j=1}^{M_i} y_{i,j}}{\sum_{i=1}^{n} M_i}$$

The standard error appropriate to the cluster sample given equal $M_i$ is estimated from the deviation among the cluster mean values

$$s_{\bar{y}}^2 = \frac{s_{zw}^2}{n}$$

And where $M_i$ varies among clusters

$$v(\bar{y}) = \frac{1}{\left(\sum_{i=1}^{n} M_i\right)^2} \cdot \frac{n}{n-1} \cdot \sum_{i=1}^{n} M_i^{\,2} \left(\bar{y}_i - \bar{y}\right)^2$$

where n = size of sample in each stratum, M = number of elements (plots) in each cluster, $y_{i,j}$ = stand volume per hectare estimated in sample plots j in cluster i.

In the Lenga forest (stratum I), 371 satellite samples were taken in the first phase and 30 samples were selected in the second phase. In the degraded Lenga forest (stratum II), 51 satellite sample were taken and 14 clusters were assessed terrestrially.

The mean values of the quotients IR II and IR, to be taken from the windows, were related to the corresponding mean stand volume values from the cluster samples. In this way the regression models for estimating the stand volume could be set up.

## C Reservoirs in Living Trees

To estimate the C reservoirs in the various compartments of the ecosystem, two plots each of 1 ha of the **two typical pristine Lenga forest structures** were set up: one of regular and one of irregular structure. Both were situated in a place of medium site quality within the Fontana and La Plata lake basin. A total of 56 sample trees from different sociological positions in the canopy were randomly selected from both stands. Their diameter at breast height (DBH) varied between 10 and 80 cm. These trees were felled in order to study their biomass using a destructive method.

The C reservoirs in living trees may be calculated using the following equation:

$$C_{LT} = [B_{BB} + B_C + B_R] * C_{cont}$$

where $C_{LT}$ = C in living trees, $B_{BB}$ = biomass in boles and main branches larger than 10 cm in diameter, $B_C$ = biomass in crowns (branches less than 10 cm in diameter plus leaves), $B_R$ = biomass in roots; $C_{cont}$ = C content of biomass.

Biomass may be determined by weight or volume and the density of its components. In this study, Lenga bole biomass was determined by volume and density and crown biomass by weight. By this method the equation to determine C contents of living trees based in the use of forest inventories is:

$$C_{LT}\ (t/ha) = V_{BB}\ (m^3/ha) * \delta_m\ (tn/m^3) * R_f * E_f * C_{cont}$$

where $V_{BB}$ = boles and main branches volume, $\delta_m$ = average density value for healthy wood, $R_f$ = reduction factor, $E_f$ = expansion factor.

## Biomass in Standing Wood and Main Branches Larger than 10 cm in Diameter

The biomass of the bole and main branches of each tree was determined based on the volume and density of the diverse wood tissues and their state of health, including: (1) healthy sapwood, (2) healthy hardwood, (3) hardwood showing early brown rot, (4) hardwood showing advanced brown rot, and (5) hardwood showing white rot.

Each tree, once felled, was initially cut 30 cm from its base. The remaining bole was then cut again into 2-m-long logs, up to a 10-cm diameter at their smaller end. Data collected by digital measurements of the surface area of each wood component and the state of health of both end-faces of the logs was computer processed. Then, using the Smallian formula, their volume was calculated. The volume of the bole and main branches of each tree resulted from the integration of the logs' volume found in this way.

In order to assess the density for each wood component and state of health, a thorough investigation was conducted, comprising samples from most of the end-faces. Basic density was determined (dry weight/damp volume). The volume of each sample was measured by the water-displacement method, and their dry weight was taken after oven-drying the samples at 105ºC, to constant weight.

## Biomass in Crowns and Roots

Crown biomass of individual trees, comprising branches less than 10 cm in diameter (and leaves), was determined by means of on-site weighing in the forest. To translate these values into dry-weight readings, their moisture content was measured using samples that were later oven-dried at the lab (105ºC, to constant weight).

Root biomass was not directly measured in this study, but estimated through an equation that, using a reduced number of trees, was adapted by Weber[35] for the Tierra del Fuego Lenga forests. This equation is:

$$Ln(B_{und}) = 7{,}9124 - 23{,}3104 * DBH^{-0{,}5} + 13{,}2689 * DBH^{-1}$$

where $Ln(B_{und})$ = underground biomass logarithm.

## C Content of Biomass

Percentage C contents of each wood component were found by the sulphuric acid – potassium dichromate digestion and colorimetric determination technique at the Soil Lab of the Forestry College in Valdivia University (Chile).

## Biomass Reduction and Expansion Factors for Lenga

Usually parts of the tissue from living trees in natural forests of Lenga suffer from brown and white rot, caused by wood-decomposing fungi[36]. In the rotting process, a reduction in the density of these tissues occurs, and $CO_2$ is consequently emitted into the atmosphere[37]. Therefore, tissue of the same kind has a variable biomass, according to the density reduction that decomposition may have caused[38]. On the other hand, in the sapwood-to-hardwood transformation process, heavy chemical substances accumulate along with lignification, ending in a small increase in density, and consequently biomass[37]. Then the net biomass of bole and main branches, measured using volume and density values, may be expressed as follows:

$$nB_{BB} = Vol_b * \delta_b + Vol_s * \delta_s + Vol_{hh} * \delta_{hh} + Vol_{ebr} * \delta_{ebr} + Vol_{abr} * \delta_{abr} + Vol_{wr} * \delta_{wr}$$

where $B_{BB}$ = net biomass in bole and main branches, $Vol_b$ = bark volume, $\delta_b$ = bark density, $Vol_s$ = sapwood volume, $\delta_s$ = sapwood density, $Vol_{hh}$ = healthy hardwood volume, $\delta_{hh}$ = healthy hardwood density, $Vol_{ebr}$ = volume of early brown rot; $\delta_{ebr}$ = density of early brown rot, $Vol_{abr}$ = volume of advanced brown rot, $\delta_{abr}$ = density of advanced brown rot, $Vol_{wr}$ = volume of white rot, $\delta_{wr}$ = density of white rot.

The net biomass in bole and main branches ($nB_{BB}$) differs from the gross biomass in bole and main branches ($gB_{BB}$), in that the latter would be the resulting biomass if rotten wood and healthy wood had the same density. $gB_{bb}$ may be calculated by multiplying volume data from forest inventories by average density values for healthy wood. In order to estimate $nB_{bb}$ using $gB_{bb}$ data, the density reduction caused by rot must be known. That ratio is called the *Reduction factor* ($R_f$) and may be calculated as follows:

$$R_f = nB_{BB} / gB_{BB}$$

Estimation of the total tree biomass (bole, crown, roots) based on inventory data requires knowledge of the ratio between $nB_{bb}$ and the total biomass, which comprises the sum of $nB_{bb}$, the biomass in the crown ($B_c$) and the biomass in the roots ($B_{und}$). This ratio is called the *Expansion factor*:

$$E_f = (nB_{BB} + B_c + B_{und}) / nB_{bBB}$$

Owing to the fact that $nB_{bb}$ and $B_c$ were determined in this study, while $B_{und}$ was estimated using Weber’s equation, separate values were obtained for the *aerial Expansion factor* ($aE_f$) – crowns – and the *total Expansion factor* ($tE_f$) – crowns plus roots.

## C in Dead Wood, O Horizon and Herbage

On the five plots of each cluster sample in both strata, the quantity of dead wood lying on the ground was recorded. The diameter (including branches up to 20 cm in diameter), length, and stage of decomposition were measured or estimated. Three stages of decomposition were distinguished: (1) intact wood, not resting on the ground; (2) barkless, heartwood decomposed/sapwood hard or heartwood hard/sapwood decomposed, trunk resting on ground; (3) heartwood and sapwood very decomposed (Fig. 4).

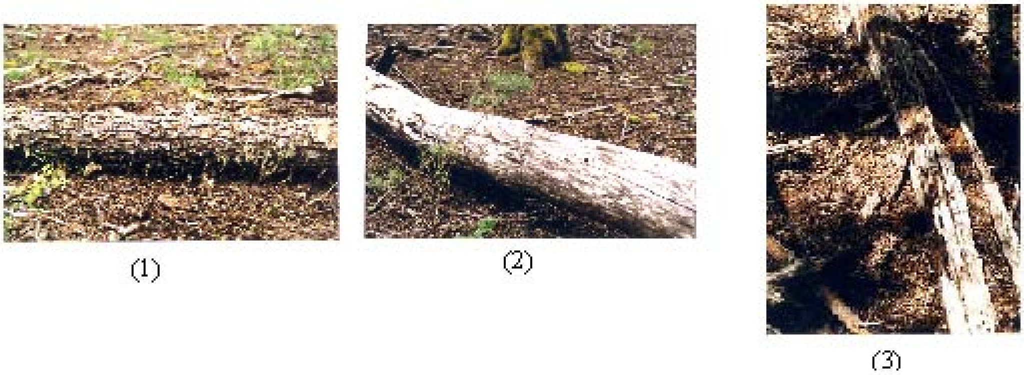

**FIGURE 4**. Stages of decomposition of dead wood.

To determine the density of the dead wood, 20 to 30 samples of each stage of decomposition were taken in the 1-ha plots of regular and irregular structure.

In order to estimate the biomass of the undergrowth, the small dead wood (<10 cm), and the C content in the O horizon (organic layer), 25 plots of 1 $m^2$ were systematically set up in each of the two stand structures (regular and irregular), using the method of Macdicken[39]. All individual young Lenga trees, shrub and herb species, the small dead wood, and the O horizon were taken from each subplot and weighed. One sample of each of the materials per subplot was taken in order to determine the water content. Deeper soil layers have not been investigated.

## RESULTS

### Forest Distribution in the Project Area

The *Nothofagus* forests cover an area of 50,275 ha, of which 44,484 ha are *N. pumilio* (Lenga) and 5791 ha *N. antarctica* (Ñire) (see Table 2). However, 2600 ha of the total forest area were burnt in 1999, when a forest fire destroyed parts of the forest on the northern shore of Lake Fontana.

Discounting the Lenga forest area in higher areas above 1150 m a.s.l., the virgin Lenga forests dominate the landscape on about 13,670 ha in the western part of the project area. The managed forest areas are in stratum 1 (potentially productive forests) included and cover approximately 4281 ha (Fig. 5).

**TABLE 2**
**Current Distribution of Forest Ecosystems in the Project Area[11]**

| Ecosystem | Area in hectares |
|---|---|
| Lenga, mean height <14 m, over 1150 m a.s.l. | 18,870 |
| Lenga, mean height >14 m, under 1150 m a.s.l. (I) | 22,850 |
| Degraded Lenga, mean height >14 m, <1150 m a.s.l. (II) | 2050 |
| Ñire | 3905 |
| Lenga* | 714 |
| Ñire* | 1886 |
| Pastures* | 260 |

* Burnt areas

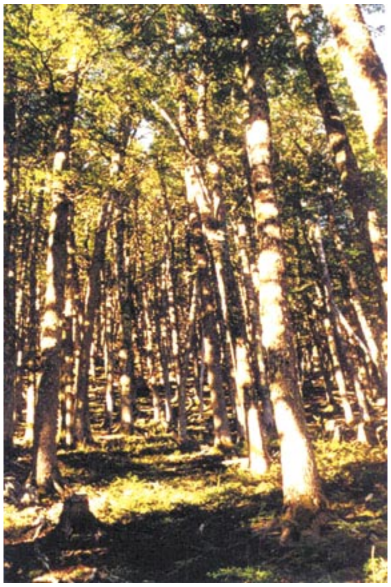

**FIGURE 5**. Lenga stand used approximately 30 years ago.

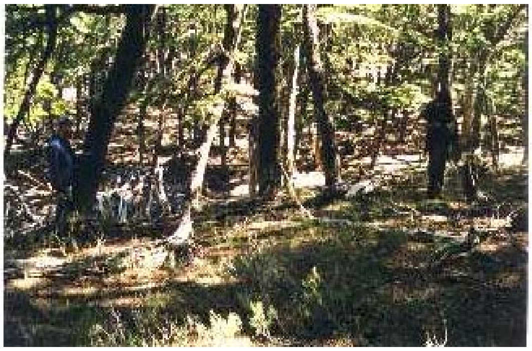

**FIGURE 6**. Degraded Lenga forests.

Stands are of various ages and harvests based on current silvicultural methods leave large amounts of dead wood on the forest floor. The majority of the harvested areas around Lake La Plata are in a strip of up to 650 m in width along the bank, as formerly the wood was floated across the lake.

Impoverished Lenga forest stands (due to forest pasture, forest fires, and timber use) cover 2050 ha (Fig. 6).

## Mean Stand Volume of Lenga (Stratum I)

In the regression analysis in the two-phase sampling, the vegetation index IRII shows a significant correlation with the stand volume values in stratum I ($r^2 = 0.28$, $F = 7.7$), but the correlation is not sufficiently high (Fig. 7). The following regression model was selected:

$$V\ (m^3/ha) = -244 + 1{,}748 \cdot IR\ II$$

Based on 371 satellite samples ($n_s$) and using the regression model, the mean stand volume per hectare was then estimated (Table 3). A value of 508 $m^3$/ha with a standard error of the estimated mean of 21 $m^3$/ha emerged. The mean stand volume per hectare from the terrestrial assessment ($n_t$) was 530 $m^3$/ha with a standard error of 28 $m^3$/ha.

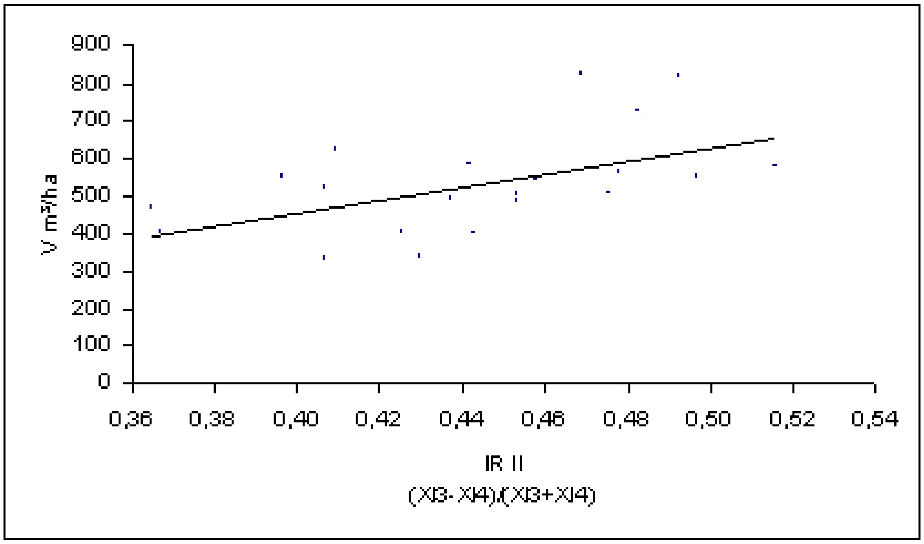

**FIGURE 7**. Regression to determine the correlation between stand volume and vegetation index IR II in Lenga forests (stratum I).

**TABLE 3**
**Comparison of the Estimate of Stand Volume Based on Two-Phase Random Sample Tests and Terrestrial-Only Cluster Samples in Lenga Forest Stratum 1**

| **Lenga (Stratum I)** | **Two-Phase Sampling ($n_t$ = 30)/($n_s$ = 371)** | **Terrestrial Cluster Sampling ($n_t$ = 30)** |
|---|---|---|
| Stand volume per hectare, $\bar{y}$ (m³) | 508 | 530 |
| Standard error of estimate, $s_{\bar{y}}$ (m³) | 21 | 28 |

With a coefficient of determination of $r^2 = 0.28$, each sample from the satellite data must be at least 12 times cheaper than the terrestrial sample (see above), in order to increase accuracy in comparison with the one-phased ground sampling at the same cost. The cost ratio (cost per sample unit in phase 2: cost per sample unit in phase 1) for the inventoried Lenga forest area is 41 to 1.

## Degraded Lenga (Stratum II)

For degraded Lenga the vegetation index IR showed closer correlation with the stand volume than the index IR II. In the regression analysis the following equation ($r^2 = 0.58$, $F = 5.5$) was selected for the two-phase sampling:

$$V\ (m^3/ha) = 497 + 25 \cdot IR - 1{,}018 \cdot IR\ II$$

The correlation between the values of IR and the stand volume details is shown in Fig. 8.

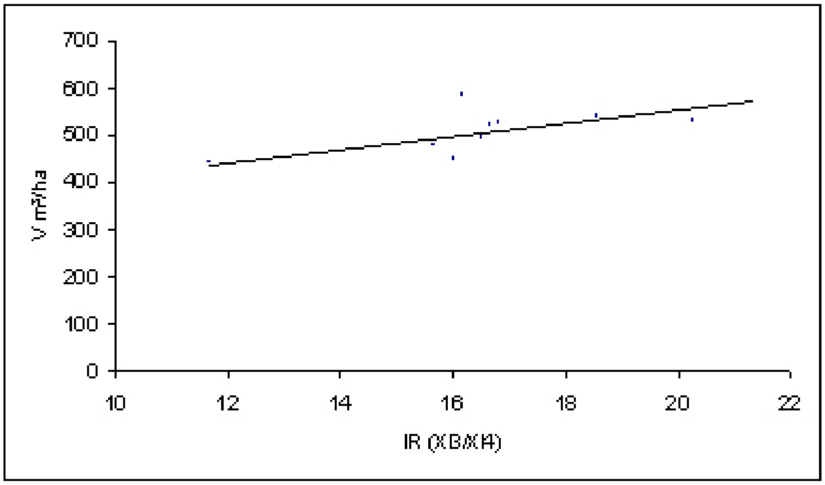

**FIGURE 8**. Correlation between stand volume values and index IR in degraded Lenga.

With the help of the mean values for IR and IR II from 51 satellite picture samples, the mean stand volume per hectare was estimated using the regression model (Table 4). A value of 486 m³/ha with a standard error of estimation of 9 m³/ha emerged. The mean stand volume per hectare emerging from terrestrial measurements was 489 m³/ha with a standard error of estimation of 27 m³/ha.

The cost ratio between terrestrial sampling and satellite sampling in this type of forest is 34 to 1. Table 5 shows a summary of the statistical values for both strata.

**TABLE 4**
**Comparison of the Stand Volume Estimate Based on Two-Phase Random Sampling and Terrestrial-Only Cluster Sampling in Lenga Stratum 2**

| **Degraded Lenga** | **Two-Phase Sampling ($n_t$ = 14)/($n_s$ = 51)** | **Terrestrial Cluster Sampling ($n_t$ = 14)** |
|---|---|---|
| Stand volume per hectare, $\bar{y}$ (m³) | 486 | 489 |
| Standard error of estimation, $s_{\bar{y}}$ (m³) | 9 | 27 |

**TABLE 5**
**Statistical Values for Stand Volume**

| **Lenga Forests** | **Stratum I** | **Stratum II** |
|---|---|---|
| Boles and main branches volume, $V_{bb}$ (m³/ha) | 508 | 486 |
| Standard error of estimation, $s_{\bar{y}}$ (m³/ha) | 21 | 9 |
| Percentage error of the 95% confidence interval | 9 | 4 |

Although the volume values for the two strata do not differ greatly, there is a significant difference in the wood quality of the trees. In stratum II approximately 50% of the trees with a DBH of over 30 cm have some rot, whereas this applies to only 34% of the trees in stratum I. There is only slight regeneration in stratum II[11].

## C Reservoirs in Lenga Trees

Table 6 shows the diameter class distribution of the 59 trees that were felled for this study. The distribution gives a representative picture of the diameter distribution in the investigated Lenga stands.

**TABLE 6**
**Diameter Class Distribution of Investigated Trees**

| DBH-class (cm) | n |
|---|---|
| 10 – 19 | 11 |
| 20 – 29 | 15 |
| 30 – 39 | 14 |
| 40 – 49 | 6 |
| 50 – 59 | 8 |
| 60 – 69 | 2 |
| 70 – 79 | 2 |
| 80 – 89 | 1 |
| **Total** | **59** |

where n = size of sample.

## Density, Expansion, and Reduction Factors

Table 7 shows average values and variation for the density of each wood component and their state of health. There are no differences in the density of healthy hardwood and that of wood with early brown rot. Advanced brown rot density is on average 35% less than the healthy hardwood density, and white rot density is 62% lower than healthy hardwood values. The bark shows the greatest density.

**TABLE 7**
**Wood Component Density ($g/cm^3$)**

| Wood Component | N | Average Density ($g/cm^3$) | SD | CV (%) |
|---|---|---|---|---|
| Bark | 464 | 0.516 | 0.0609 | 11.81 |
| Sapwood | 510 | 0.437 | 0.0365 | 8.35 |
| Healthy hardwood | 289 | 0.475 | 0.0356 | 7.49 |
| Early brown rot hardwood | 377 | 0.481 | 0.0370 | 7.69 |
| Advanced brown rot hardwood | 337 | 0.307 | 0.0791 | 25.78 |
| White rot hardwood | 130 | 0.177 | 0.0680 | 38.08 |

where N = size of sample, CV = coefficient of variation, SD = standard deviation.

**Reduction factors**—$R_f$ values vary considerably among the studied Lenga trees, all diameter classes considered. For larger individual trees, especially over 60 cm in diameter, they do not exceed the 0.9 mark (Fig. 9).

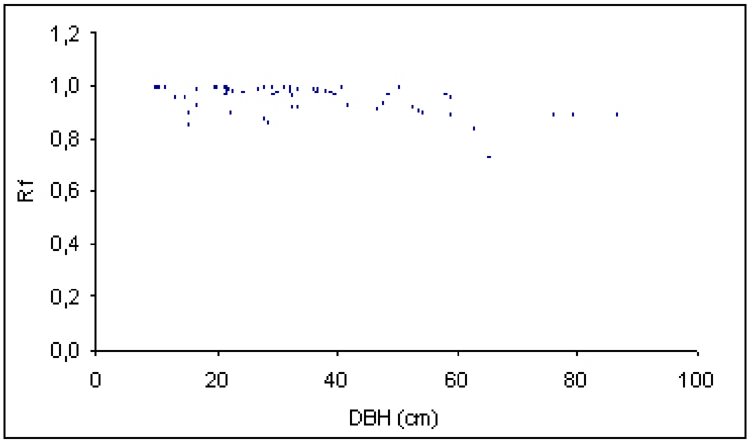

**FIGURE 9**. Scattergram of reduction factors as a function of DBH.

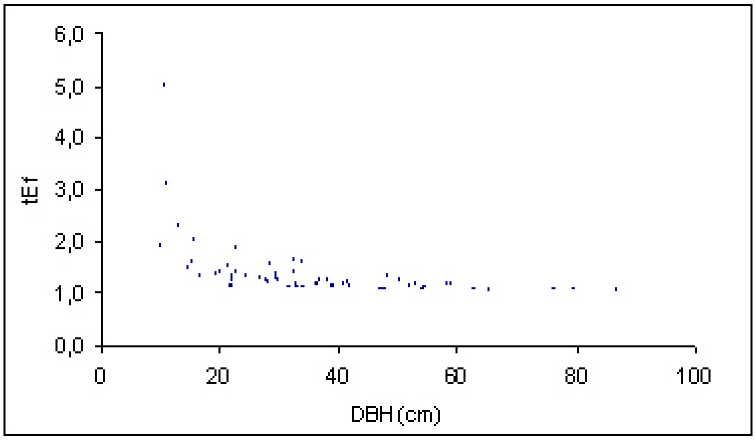

**FIGURE 10**. Scattergram of expansion factors as a function of DBH.

**TABLE 8**
**Diameter-Class Values for Biomass Factors**

| **DBH** | **Rf** | | | **Aerial Ef** | | | **Total Ef** | | |
|---|---|---|---|---|---|---|---|---|---|
| **(cm)** | **n** | $\bar{x}$ | **CV (%)** | **n** | $\bar{x}$ | **CV (%)** | **n** | $\bar{x}$ | **CV (%)** |
| 10 - 20 | 40 | 0.965 | 4 | 11 | 2035 | 56 | 11 | 2550 | 59 |
| 21 - 40 | | | | 29 | 1319 | 14 | 29 | 1568 | 15 |
| >41 | 19 | 0.915 | 7 | 19 | 1154 | 7 | 19 | 1294 | 8 |

**Expansion factors**—$E_f$ also show great variation among the studied Lenga trees, especially for smaller individual trees. Values for $E_f$ tend to reach 1 for the larger trees (Fig. 10).

The authors suggest stratification of these factors, owing to a relatively high dispersion in their values. These simplified coefficients are noted in Table 8.

## C Content of the Biomass

Analysis of the C content of each wood component did not show any significant differences, values remain close to 45% (Table 9).

**TABLE 9**
**C Content of Wood Components with Different States of Health**

| Wood Component | Ccon [%] |
|---|---|
| Sapwood | 44.6 |
| Healthy hardwood | 45.2 |
| Early brown rot hardwood | 45.0 |
| Advanced brown rot hardwood | 47.0 |
| White rot hardwood | 45.1 |

## C in Dead Wood

The high proportion of dead wood reflects the dynamics of natural forests in which all dying wood remains on the spot and is subject to natural decomposition. As can be seen from the Table 10, the majority of the dead wood was allocated to decomposition stage 3.

**TABLE 10**
**Volume and C Content of Dead Wood (Diameter >20 cm)**

| | Stratum I | | | Stratum II | |
|---|---|---|---|---|---|
| **Stage of Decomposition** | **V m³/ha** | **Density (t/m³)** | **C (t/ha)** | **V m³/ha** | **C (t/ha)** |
| 1 | 3 | 0.423 | 0.5 | 6 | 1.1 |
| 2 | 25 | 0.317 | 3.5 | 34 | 4.8 |
| 3 | 101 | 0.233 | 10.5 | 72 | 7.5 |
| **Total** | 129 | | 14.5 | 112 | 13.4 |
| | $s_{\bar{y}\%} = 7$ | | | $s_{\bar{y}\%} = 2.8$ | |

The mean values are below those given by Loguercio[40]: in the irregularly structured stands there are 188 m³ dead wood per hectare and in the regularly structured stands 153 m³ dead wood per hectare. One reason for this is that the values in the table refer to dead wood over 20 cm in diameter, whereas in the calculation of the dead wood volume by Loguercio[40], branches from 10 cm diameter are included.

## C Content of O Horizon, Lenga Young Trees, Shrubs, and Herbage

Table 11 shows the biomass and the C content for both stands, one of regular and one of irregular structure. Because of the dense regeneration in the irregularly structured stand, the biomass for this area is significantly higher. The other components show similar values for biomass and C content in both stands.

An average C content in the vegetation on the ground and O horizon of 18.2 t/ha may be assumed in the investigated areas.

**TABLE 11**
**Biomass and C Content in O Horizon, Herbage, and Young Trees**

| Structure | Para-meter | Biomass (t/ha) | | | | | C (t/ha) | | | | |
|---|---|---|---|---|---|---|---|---|---|---|---|
| | | Lenga Young Trees | Shrubs | Dead Wood <10 cm | O Horiz. | Total | Lenga Young Trees | Shrubs | Dead Wood <10 cm | Ao Horiz. | Total |
| Irregular | $\overline{x}$ | 15.7 | 2.1 | 7.9 | 18.5 | 44.1 | 7.8 | 1.0 | 3.9 | 9.2 | 21.9 |
| | S | 23.8 | 1.6 | 6.7 | 5.9 | 26.9 | 22.6 | 1.5 | 6.2 | 5.6 | 25.1 |
| Regular | $\overline{x}$ | 3.7 | 1.5 | 8.7 | 15.5 | 29.4 | 1.9 | 0.7 | 4.3 | 7.7 | 14.6 |
| | S | 7.5 | 0..9 | 6.3 | 3.4 | 8.8 | 6.9 | 0.9 | 6.0 | 6.0 | 8.2 |

## Total C in Living Trees of Project Area

The C content in the living trees in the potentially productive Lenga forests is 136 t C/ha. The C content in the dead wood in these forests is 10.6% of the C content of the living trees. It is estimated that the O horizon and shrub layer contain 13.3% of the C content accumulated in the living trees (see Table 12).

**TABLE 12**
**Total Amount of C in the Project Area**

| Current status | Unit | Lenga protection forest | Potentially productive lenga forest | Degraded Lenga | Ñire | Burnt forest | Total / average |
|---|---|---|---|---|---|---|---|
| Area | ha | 18,870 | 22,850 | 2,050 | 3,905 | 2,600 | 50,275 |
| Volume | Standing gross volume in $m^3$ / ha (>10cm) | 350 | 508 | 486 | 100* | 200* | 400.2 |
| Wood density | $t/m^3$ | 0.46 | 0.46 | 0.46 | 0.5 | 0.46 | 0.5 |
| Expansion factor | | 1.40 | 1.40 | 1.37 | 1.25 | 1.40 | 1.4 |
| Reduction factor | | 0.93 | 0.93 | 0.93 | 1.00 | 0.93 | 0.9 |
| C content | t/t dryweight | 0.45 | 0.45 | 0.45 | 0.45 | 0.45 | 0.4 |
| C in living trees | t/ha | 93.3 | 136.2 | 126.3 | 28.1 | 53.6 | 107.4 |
| C in dead wood (d > 20 cm) | t/ha | 10 | 14.5 | 13.4 | 1.0 | 25.0 | 12.3 |
| C in herbage, organic layer | t/ha | 18.2 | 18.2 | 15** | 10 | 8 | 16.9 |
| C per hectare in total | t/ha | 121.5 | 168.9 | 154.7 | 39.1 | 86,6 | 136.2 |
| Total C in living dendromass | 1000 t | 1,761 | 3,113 | 259 | 110 | 139 | 5,382 |
| Total $CO_2$ equivalents in living dendromass | 1000 t | 6,457 | 11,413 | 950 | 402 | 511 | 19,733 |
| Total C | 1000 t | 2,293 | 3,860 | 317 | 153 | 225 | 6,848 |
| Total $CO_2$ equivalents | 1000 t | 8,408 | 14,153 | 1,163 | 559 | 826 | 25,109 |

* No inventories have been implemented in Lenga protection forest, Ñire stands, and in burnt areas. The standing stock used to calculate the C reservoirs of this part of the project area is therefore based on an estimation.

** Estimation

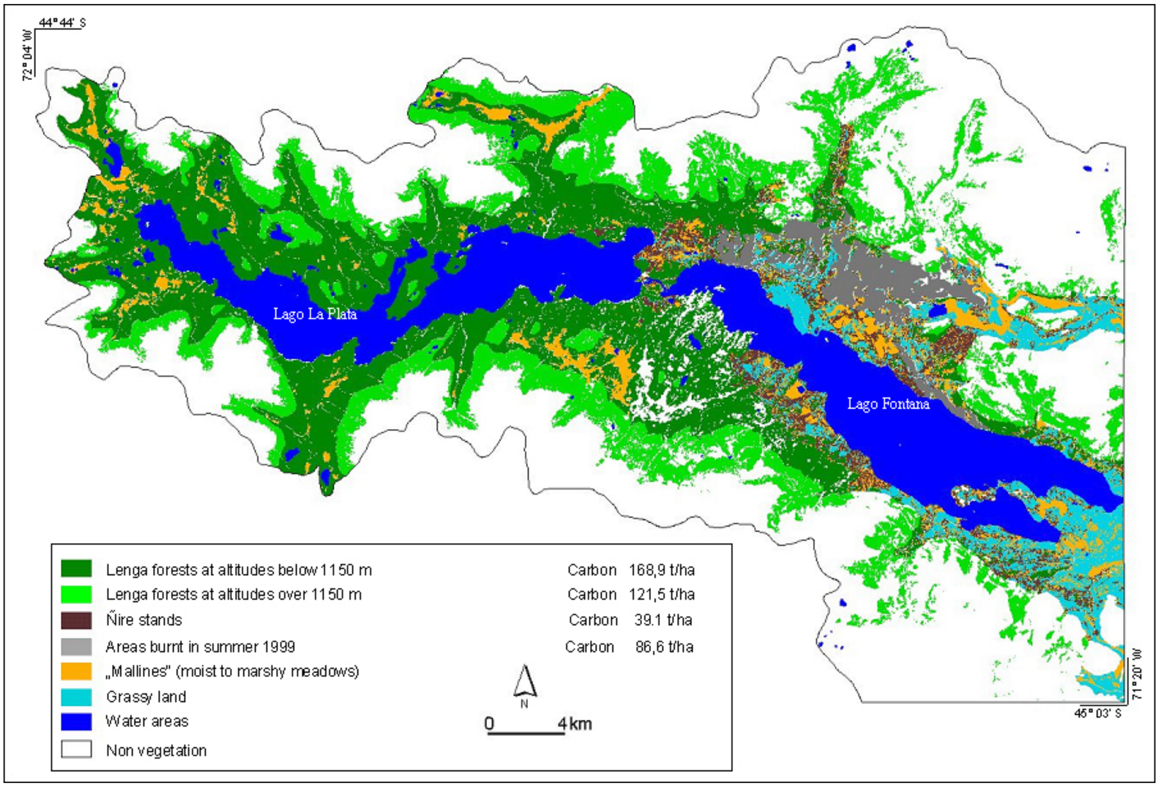

**FIGURE 11**. Mean volume of C in the project area.

The total quantity of C in the forests in the project area — excluding C in deeper soil layers — is 6.848 Mio.t C. Fig. 11 shows the mean C volume in the project area.

## DISCUSSION

Knowing about their considerable influence on global climate change, the international climate debate focused attention on forests and forestry, among other things. However, there is a lack of regional data describing the C reservoirs and flows in detail. This paper contributes to the partial closing of this data gap with an investigation at regional scale of the C reservoirs in a South American forest ecosystem.

The investigation first of all put emphasis on the area estimation. To map the area and the vegetation types, SPOT data have been used. Due to the experience of this study, SPOT data, including auxiliary information (e.g., derived from a digital elevation model), are suitable for classifying the vegetation and stratifying the forest types. The method allows for the vegetation and C reservoirs to be mapped at a scale of 1:50,000. Up to now, maps have only been available for the region with a scale of 1:250,000.

There is a plethora of local scale models that describe the cycle of C through forest ecosystems and how these processes react to natural and manmade effects[41]. Due to the spatial nature of many models it has been difficult to scale up from the local to regional scale[42]. This has led to the development of scale-independent deterministic models (e.g., Forest BGC, TCX model, [43,44]) that use climatic data and the spatial, temporal and spectral capabilities of remote sensing[45]. The input variables of the applied scale-independent model (to estimate the volume of the stands) were extracted from satellite data. We believe that this method, based on a solid statistic, allows for investigations in temperate forest ecosystems with insufficient information.

For the calculation of costs, both the relevant proportion of costs for acquiring the SPOT 4 scene required for the two-phase sampling, and the costs for the use of the image-processing equipment were taken into account. The cost ratio of terrestrial and satellite samples in the two-phase sampling method was between 34 and 41 to 1 in this study. Scheer et al.[29] established a ratio of 13 to 1 for applying SPOT data in Slovakia. The relative costs when using medium-scaled aerial photography in the first phase are between 5 and 15 to 1[31]. Therefore the two-phase sampling applying satellite data and terrestrial permanent cluster samples represents an efficient alternative for estimating the stand volume for large areas and monitoring, especially in inaccessible forest areas.

In terms of ease of measurement, it is more convenient to deduce the auxiliary variables (vegetation indices) from satellite data than to measure the auxiliary variables from aerial photography samples. In this the work involved is independent of the number of satellite samples, as the deduction is made automatically. Like permanent aerial photography samples[46], permanent satellite picture samples may also be taken. These, together with the established permanent cluster samples, allow more accurate estimates of the change in vegetation and stand volume.

The value of the reduction factor hovers at around 0.93, and shows a tendency to decrease for higher diameters. The expansion factors decrease with a higher diameter in breast height. This fact is confirmed by Brown[47] for forests in the temperate zone. Assuming, that rot increases with DBH, Weber[35] developed a theoretical pattern in order to estimate biomass reduction in Lenga. In his pattern, the reduction factor varies betweeen 0.998 for a DBH smaller than 30 cm and 0.798 for a DBH larger than 80 cm. These values are slightly below the results found in this study. The expansion factor decrease with a higher diameter at breast height, ranging from 2.550 for a DBH between 10 and 20 cm to 1.294 for DBH < 41 cm. These results are confirmed by Brown[47] for forests in the temperate zone. Richter and Frangi[48] reported 1.535 quotient between total and bole biomass in Tierra del Fuego Lenga forest.

The C content in protection forests (living trees) is assumed to be 93 t C/ha. This C content is smaller than in potentially productive forests because of the lesser stand volume (s. Table. 12). Although further investigation is necessary for this, it is to be assumed that future use of the forests (forestry, grazing livestock) is out of question. Further studies are necessary to determine the C reservoirs of the Ñire stands and the burnt areas. A stratification of these stands using SPOT data would lead to greater accuracy in the estimation of the biomass. Especially Ñire forests are used for forestry and as pastures. In addition, they are also at high risk from fires, so that monitoring should be carried out at frequent intervals.

The C content calculated at 136 t C/ha in potentially productive Lenga forests (living trees) is below the value of 202 t C/ha derived by Weber[35] in Tierra del Fuego. The stand volume estimated there was however 580 to 779 m³/ha, depending on the structure, and therefore on average some 180 m³/ha more than in this investigation. Richter and Frangi[48] also established a value of 239 t C/ha in virgin Lenga forests. Here it must be taken into account that around 40% of the area in stratum II of the Lenga forests defined as productive forest has been formerly logged. In some stands, intervention was undertaken which was not sustainable. The average stand volume of the clusters in virgin forests is around 554 m³/ha and that of the used stands is 471 m³/ha. On average a stand volume of 508 m³/ha is estimated for this stratum. Determining changes in the area is decisive in monitoring the C reservoirs.

Although the importance of the ground vegetation as a C reservoir is with a value of 0.4 t C/ha very slight in Tierra del Fuego[35], the ground vegetation in the investigated area made a significant contribution to the total stand volume. The comparably high C reservoir in the ground vegetation of the La Plata area, which sums up to a mean of 11.5 t C/ha, is due to the fact that the vegetation period as well as the growing conditions in the project area are much more favourable than 1100 km south in Tierra del Fuego.

A similar situation can be stated concerning the C reservoirs in dead wood. Whilst Weber[35] investigated a stand volume of 34.4 t C/ha in virgin Lenga forest in Tierra del Fuego, the longer decaying period of the La Plata region results in a reduced C reservoir in dead wood of about 14 t C/ha.

We would be pleased to compare our results with similar studies to improve our methods and to foster a discussion about the topic.

## ACKNOWLEDGEMENTS

This work has been initiated under the auspices of PRIMAKLIMA – weltweit – e.V., Ikenstrasse 1B, 40625 Duesseldorf, Germany. Tel. 0049 211 295419; Fax. 0049 211 2913682, e-mail: prima-klima@user.ecore.net.

The authors are greatful to the European Union and the German Ministry of Environment for financing the PRIMAKLIMA-Project „Forestry Options for Carbon Sequestration", which served to analyse the project area scientifically.

The authors would like to thank Jorge A. Miglioli, Bernhard Schlamadinger, Martin Schröder, and Tessa Feller for their helpful scientific and linguistic comments.

This paper reflects the views of the authors and not necessarily the views of the institutions with which they are affiliated.

## ADDITIONAL REFERENCES

Apps, M.J. and Kurz, W. (1991) Assessing the role of Canadian forests and forest sector activities in the global carbon cycle. *World Resour. Rev*. **3**, 333–344.

BML. (1990) Bundeswaldinventur, Band I: Inventurbericht und Übersichtstabellen für das Bundesgebiet nach dem Gebietsstand vor dem 03.10.1990 einschließlich Berlin (West). Band II: Grundtabellen für das Bundesgebiet nach dem Gebietsstand vor dem 03.10.1990 einschließlich Berlin (West). Bundesministerium für Ernährung, Landwirtschaft und Forsten (Hrsg.). Bonn.

BML. (1994) Der Wald in den neuen Bundesländern. Eine Auswertung vorhandener Daten nach dem Muster der Bundeswaldinventur. Bundesministerium für Ernährung, Landwirtschaft und Forsten (Hrsg.). Bonn.

Böswald, K. (1995) Wald und Forstwirtschaft im regionalen Kohlenstoffhaushalt Bayerns. *Allg. Forst Z.* **6**, 291–295.

Böswald, K. (1996) Zur Bedeutung des Waldes und der Forstwirtschaft im Kohlenstoffhaushalt - Eine Analyse am Beispiel des Bundeslandes Bayern. Schriftenreihe der Forstwissenschaftlichen Fakultät der Universität München und der Bayerischen Landesanstalt für Wald und Forstwirtschaft, Band 159.

Böswald, K. and Wierling, R. (1997) Wald und Forstwirtschaft Niedersachsens im Kohlenstoffhaushalt. Gutachten für das Niedersächsische Ministerium für Ernährung, Landwirtschaft und Forsten. Aus dem Walde, Heft 50.

Böswald, K. (1998a) The present role of german forests in the global carbon budget and options to it`s increase. In Climate Change Mitigation and European Land Use Policy. CAB International.

Böswald, K. (1998b) Present and Future Options of Forests and Forestry for CO2-Mitigation in Germany. Proceedings: Carbon Mitigation Potentials of Forests and Wood Industry. Workshop, Munich Freising, June 19–21, 1996. Springer-Verlag.

Bosshard, H.H. (1984) Holzkunde (Bd. 2 und 3); Birkhäuser Verlag, Basel, Boston, Stuttgart.

Burschel, P., Kürsten, E., and Larson, B.C. (1993) Die Rolle von Wald und Forstwirtschaft im Kohlenstoffhaushalt - eine Betrachtung für die Bundesrepublik Deutschland. Schriftenreihe der Forstwissenschaftlichen Fakultät der Universität München und der Bayerischen Forstlichen Versuchs- und Forschungsanstalt München (Hrsg.). München, 135 p.

Daube, W. (1883) Elementare Zusammensetzung und Aschegehalt von Splint- und Kernholz. *Forstl. Blätter* **20**, 392.

Dauber, E. and Zenke, B. (1978) Potential forstlicher Reststoffe (Waldabfälle). Bd. 1. FB Forstwissenschaft der Ludwig-Maximilians-Universität München.

Droste zu Hülshoff, v. B. (1970) Struktur, Biomasse und Zuwachs eines älteren Fichtenbestandes. *Forstwiss. Centralbl*. **89**, 162–171.

Ellenberg, H., Mayer, R., and Schauermann, J. (1986) Ökosystemforschung – Ergebnisse des Sollingprojektes 1966–1986. Verlag Eugen Ulmer, Stuttgart, 507 p.

Fengel, D. and Wegener, G. (1984) *Wood - Chemistry, Ultrastructure, Reactions*. Walter de Gruyter Verlag, Berlin, 613 p.

Fiedler, F. (1986) Die Dendromasse eines hiebsreifen Fichtenbestandes. *Beitr. Forstwirtsch*. **20(4),** 171–180.

Fogel, R. (1983) Root turnover and productivity of coniferous forests. *Plant Soil* **71**, 75–85.

Gayer, K. and Fabricius, L. (1935) Die Forstbenutzung, 13. Aufl., Berlin.

Gottlieb, E. (1883) Untersuchung über die elementare Zusammensetzung einiger Holzsorten in Verbindung mit calorimetrischen Versuchen über ihre Verbrennungsfähigkeit. *J. Prakt. Chem*. **28(2),** 385–421.

Grundner, F. and Schwappach, A. (1952) *Massentafeln zur Bestimmung des Holzgehaltes stehender Waldbäume und Waldbestände.* Verlag Paul Parey, Hamburg.

Gustavsson, L., Karajalainen, T., Marland, G., Savolainen, I., Schlamadinger, B., and Apps, M. (1998) Project-Based Greenhouse Gas Accounting – Guiding Principles with Focus on Baselines. http://www.joanneum.ac.at/iea-bioenergy-Task25.

Hakkila, P. (1972) Harvesting of stumps and roots. *Commun. Inst. For. Fenn*. **77(1),** 1–71.

Holstener-Jörgensen, H. (1959) Investigations of root systems of oak, beech, and Norway spruce on ground-water-affected moraine soils with a contribution to elucidation of evapotranspiratioin of stands. *Forstl. Forsögsväs. Dan*. **25,** 227–287.

Knigge, W. and Schulz, H. (1966) *Grundriss der Forstbenutzung*. Verlag Paul Parey, Hamburg.
Köstler, J.N., Brückner, E., and Bibelriether, H. (1986) *Die Wurzeln der Waldbäume*. Verlag Paul Parey, Hamburg.
Kreutzer, K. (1976) Der Einfluß der Wirtschaftsführung auf den Nährstoffhaushalt mitteleuropäischer Wälder. Konferenz SK 2, Elmia Jönköping 20–38.
Maclaren, J.P. (1996) Plantation forestry - its role as a carbon sink: conclusions from calculations based on New Zealand`s planted forest estate. In *Forest Ecosystems, Forest Management and the Global Carbon Cycle*. Apps, M.J. and Price, D.T., Eds. NATO-ASI Series I, Vol. 40. Springer-Verlag, Heidelberg. pp. 279–292.
Maclaren, J.P., Wakelin, S.J., and Te Morenga, L. (1996) Plantation forestry in New Zealand; concepts, methodologies and recent calculations. In Greenhouse Abatement Measures: No Regrets Action Now. Proceedings of 1995 Invitation Symposium, Australian Academy of Technological Science and Engineering.
Michaelowa, A., Greiner, S., and Dutschke, M. (2001) Latest State of Discussion on Crucial Issues Concerning CDM and Forestry. Unpublished study for PrimaKlima – weltweit – e.V., Duesseldorf.
Ovington, J.D. (1957) Dry-matter protection by Pinus sylvestris. *Ann. Bot.*, N.S. **21(82),** 287–314.
Pellinen, P. (1986) Biomasseuntersuchungen im Kalkbuchenwald [Dissertation]. Universität Göttingen.
Planetary Visions Limited (2000) Gran Atlas Clarín 2000. Satellite Imagemap © 1999. http://www.planetaryvisions.com.
Price, D.T., Mair, R.M., Kurz, W.A., and Apps, M.J. (1996) Effects of forest management, harvesting and wood processing on ecosystem in carbon dynamics: a boreal case study. In *Forest Ecosystems, Forest Management and the Global Carbon Cycle.* Apps, M.J. and Price, D.T., Eds. NATO-ASI Series I, Vol. 40. Springer-Verlag, Heidelberg. pp. 279–292.
Raisch, W. (1983) Bioelementverteilung in Fichtenökosystemen der Bärhalde (Südschwarzwald). *Freiburg. Bodenkd. Abh*. 11.
Rohner, M. and Böswald, K. (2001) Forestry development scenarios: timber production, carbon dynamics in tree biomass and forest values in Germany. *Silva Fenn*. **35**(**5**), 277–297.
Süchting, H. (1943) Über Zersetzungs- und Humusbildungsvorgänge bei Waldstreu-, AO-Humusarten und Vergleichs- und Modellstoffen. *Bodenkd. Pflanzenernähr*. **32/77(4/5),** 243–295.
Trendelenburg, R. (1939) *Das Holz als Rohstoff*. J.F. Lehmanns Verlag, München, 435 p.
Ulrich, B., Mayer, R., and Heller, H. (1974) Data analysis and data synthesis of forest ecosystems. Report of International Woodlands Workshop. *Göttinger Bodenkd. Ber*. **30,** 495.
UNFCCC (1992) United Nations Framework Convention on Climate Change, http://www.unfccc.org/-resource/conv/index.html.
Volz, K.-R., Böswald, K., and Dinkelaker, F. (1996) Forstpolitik - Entwicklungen und Perspektiven. In Nachhaltige Land- und Forstwirtschaft -Expertisen. Veröffentlichungen der Akademie für Technikfolgenabschätzung. Springer-Verlag, Berlin.
Vorreiter, L. (1943) Handbuch für Holzabfallwirtschaft. 2. Aufl., Neudamm.
Vyskot, M. (1976) Tree story biomass in lowland forests in South Moravia. Academia, Praha (86)10, 166.
Vyskot, M. (1981) Biomass of the tree layer of a spruce forest in the Bohemian Uplands. Academia, Praha.
Weber, R. (1881) Vergleichende Untersuchungen über die Ansprüche der Weißtanne und Fichte and die mineralischen Nährstoffe des Bodens. *Allg. Forst Jagdztg*. **57**, 1.
